# The Prevalence and Characteristics of Intrafamilial Child and Adolescent Homicides in Greece over 11 Years (2010–2020)

**DOI:** 10.3390/children10111783

**Published:** 2023-11-04

**Authors:** Maria Tsellou, Flora Bacopoulou, Panagiotis Ferentinos, Eric Baccino, Laurent Martrille, Stavroula Papadodima

**Affiliations:** 1 Department of Forensic Medicine and Toxicology, National and Kapodistrian University of Athens, 11527 Athens, Greece; mtsellou@med.uoa.gr; 2 Center for Adolescent Medicine and UNESCO Chair in Adolescent Health Care, First Department of Pediatrics, School of Medicine, National and Kapodistrian University of Athens, Aghia Sophia Children’s Hospital, 11527 Athens, Greece; fbacopoulou@med.uoa.gr; 3 Affective Disorders and Suicide Unit, 2nd Department of Psychiatry, “Attikon” University General Hospital, School of Medicine, National and Kapodistrian University of Athens, 12462 Athens, Greece; pferentinos@med.uoa.gr; 4 EDPFM, Department of Forensic Medicine, University of Montpellier, CHU Montpellier, F-34000 Montpellier, France; e-baccino@chu-montpellier.fr (E.B.); laurent.martrille@chu-montpellier.fr (L.M.)

**Keywords:** child, adolescent, homicide, filicide, infanticide, domestic violence, psychotic

## Abstract

Intrafamilial child/adolescent homicide is the murder of a child/adolescent by one or more family members. This study delves into the medical and sociological consequences of child homicide, shedding light on the broader impact beyond individual families, which extends into the local community. Two Internet search engines and the search engines of major national news websites were surveyed to identify the number of intrafamilial child/adolescent homicide cases that occurred in Greece from January 2010 to December 2020. Over the study period, 34 victims of intrafamilial child/adolescent homicides were identified. The above deaths reflect an intrafamilial child/adolescent homicide rate of 0.15 homicides per year per 100,000 inhabitants. Most of the perpetrators (51.4%) were male, and the victims were equally divided into males and females. The ages of the perpetrators ranged from 13 to 61 years, and the ages of the victims ranged between 0 and 17 years. Most perpetrators (54.5%) had a previous psychiatric history and in many cases, they committed (33.3%) or attempted (15.2%) suicide after the homicide. The most common method of homicide was strangulation (usually combined with suffocation) (25%), followed by abandonment (15.6%). The most commonly reported motives were spousal revenge (26.5%) and psychotic disorders (26.5%). Raising awareness for intrafamilial child and adolescent homicide is of the utmost importance for the prevention of this dreadful phenomenon.

## 1. Introduction

Child homicide is probably the most heinous crime as it basically represents the most extreme form of violence directed toward children [1,2] This article delves into the medical and sociological consequences of child homicide, shedding light on the broader impact beyond individual families, which extends into the local community. Approximately 95,000 homicides of children are reported annually worldwide, and the risk of a child to be murdered clearly depends on age, gender, and country of residence [3].

According to data derived from the Center for Disease Control and Prevention (CDC) of the United States of America, the risk of homicide for children is greatest on the day of their birth [4]. This risk remains high during the first year of life [2,5,6] and decreases gradually, particularly in adolescence [7,8]. During their infancy, children exhibit increased susceptibility to homicidal acts due to their physiological fragility, dependency on parental caregivers, and their limited capacity to escape or defend against acts of aggression [9]. Conversely, middle childhood (ages 5–12) represents a phase with a relatively lower risk of child homicide when compared to younger (0–4 years) or older (13–17 years) children [2,10].

Children can be killed either by family members or by non-family members. Due to the different dynamics of these two types of killings, different terminology is used. Intrafamilial child homicide refers to the tragic act of a child’s life being taken by a family member or members. Despite the common perception of families as secure settings for children, it is disheartening to note that the majority of child homicides occur at the hands of one of the child’s parents [11]. The homicide of a child committed by a parent or parental figure(s), including guardians and step-parents, is called “filicide” (filia/filius means own daughter/son in Latin, and occidere is the Latin word for homicide or manslaughter) [12]. Another term used for killing family members is family annihilation or familicide, both of which mean the murder of all family members followed by the suicide of the perpetrator [11].

Filicide has existed since the dawn of recorded human history. In ancient Greece, Plato and Aristotle encouraged the murder of weak or malformed newborns. In ancient Rome, the killing of females, illegitimate offspring, and children deemed “surplus” was routinely practiced and rarely questioned. A father had the right to kill his own children as part of patria potens [12,13,14]. In the early Christian years, the sin of “overlaying”, the suffocation of a child by laying on top of it, was considered minor, so mothers may have committed filicide and then claimed that the child was accidentally suffocated in bed. In Judeo-Christian Europe, the profound religious beliefs against illegitimate children tacitly encouraged the killing of infants [15]. In Greek mythology, Medea killed her two sons to take revenge on their father, Jason, who had abandoned them [16]. Hercules murdered his wife and children in a state of confusion and rage [17]. The symbolism of these myths is still used nowadays to describe similar crimes. In literature, the term “Medea“ is employed to depict mothers who have resorted to filicide, a deeply tragic act, as a form of vengeance against their spouses. Fathers are often portrayed as stereotypes for committing filicide driven by rage, which is akin to the tragic example of Hercules.

Younger children (under 5 years of age) have been reported to face an elevated risk of being killed by a parent or a parental figure [2,18] as at this age, they spend most of their time with their primary caregivers. Middle-childhood-aged children (up to 12 years old) are more “protected” because their daily activities, such as attending school, keep them away from their caregivers for longer periods of time and reduce the risk (and the opportunity) of being fatally abused [19]. On the other hand, teenagers have a higher risk of being killed by extrafamilial contacts [20].

A global study in 44 countries found that parents had committed 56.5% of child homicides. Most homicides of children committed by a parent were reported in high-income countries (64.2%) and in the East Asia–Pacific Region (61.7%). The majority of children killed by their parent(s) (77.8%) were under the age of 1 year [21]. Assuming that a number of parents who commit filicide manage to conceal their heinous act, the actual rate of filicides remains unknown [22]. Therefore, filicides are believed to be underreported, especially when infants are involved [23].

In Greece, all cases of sudden and violent deaths are submitted to a complete autopsy. Suspicious or obviously violent deaths of children and adolescents are fully investigated through autopsy, toxicological examination, and, when necessary, histological examination. The autopsies may be performed by forensic services/departments belonging to the Ministry of Justice, Universities, or hospitals. The official recording of the cause of death according to age and sex is performed by the Hellenic (Greek) Statistical Service (ELSTAT). According to data from the ELSTAT, a percentage of 5.1% (60/1187 cases) of homicides were committed against children (or adult adolescents) aged 0–19 years old during the time period 2010–2019 [24]. Further details, however, regarding the circumstances of homicides (for example, intrafamilial or extrafamilial) are not officially recorded.

To our knowledge, no official data concerning intrafamilial child homicide in Greece have been published. Therefore, the aim of this study was to assess the prevalence of intrafamilial child and adolescent homicides in Greece during the past 11 years, to identify the circumstances under which they occurred and the profiles of the perpetrators and the victims, and to describe the dynamics of this type of homicide.

## 2. Materials and Methods

Mortality data from the Hellenic (Greek) Statistical Authority (ELSTAT) for the period of 2010–2020 were analyzed to determine the number of child and adolescent homicides, as well as the age and sex of the victims. However, the specific details surrounding these homicides were not available in the official mortality records of the ELSTAT. Additionally, the collection of data on child homicides proved challenging due to the decentralized nature of information across various police departments in Greece, as opposed to a centralized repository within a police record division.

In order to gather information regarding the specific circumstances surrounding child homicides, a comprehensive survey was conducted using popular Internet search engines such as Google and Yahoo, in addition to the search engines of major national news websites. The research involved the utilization of both Greek and English keywords and phrases relating to child homicide, including searches such as “killing of a 5-year-old child”, “murder of a 7-year-old girl”, “father shooting his daughter”, “family murders”, “infanticide”, “child homicide”, “child murder”, and various other similar combinations. By employing this method, all cases of child homicide reported in the official mortality records of the ELSTAT were successfully identified through Internet searches, ensuring the retrieval of cases involving children of the same age and gender for each year.

The cases of child homicides committed by parents or close family members, such as siblings and uncles/aunts, were selected from the total child homicide cases gathered through our online research. Details about the circumstances of these intrafamilial child homicides were documented based on mass media coverage, offering clearer insights into these crimes. For the purpose of this study, filicide cases were classified using Bourget and Bradford’s classification system [25] as it was modified by Liem, who added the subcategory of “psychotic filicides” [26]. The data set included (a) information about the perpetrators’ sex, age, marital status; (b) the relationship between the perpetrator and the victim (c); the method used for committing homicide; and (d) the apparent motivation or reason that could explain the homicide.

### Statistical Analysis

Absolute and relative frequencies were used for a descriptive analysis. Associations between variables were assessed using Fisher’s exact test or a Monte Carlo simulation when the chi-square test assumption was violated. The total number of the sample was assumed to be the total number of victims, though two homicides were double. In the analysis of associations, a homicide executed by both parents was excluded.

The statistical significance level was set at 0.05 (5%). The statistical analysis was performed using SPSS version 25.0 (IBM Corp. Released 2017. IBM SPSS Statistics for Windows, Version 25.0. Armonk, NY, USA: IBM Corp).

## 3. Results

**Figure 1:**
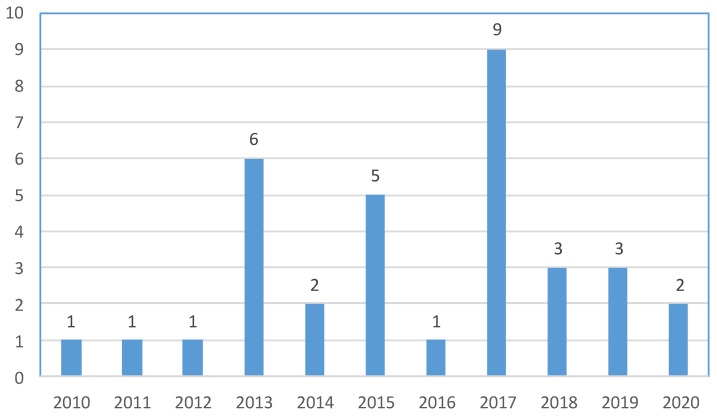
Number of intrafamilial child/adolescent homicides in Greece during the period 2010–2020.

During the 11-year study period, 34 child and adolescent victims of intrafamilial homicide were identified. The above deaths reflect an intrafamilial child/adolescent homicide rate of 0.15 homicides per year per 100,000 inhabitants. The number of intrafamilial child/adolescent homicides per year ranged from 0 to 9 (with a mean of 3.1), with no apparent trend over time (Figure 1).

The sociodemographic features of the perpetrators and the victims are shown in Table 1. There were 33 perpetrators as in one case, the victim (a 3-year-old boy) was killed by both of his parents. In two cases, one perpetrator (male in both cases) killed two children.

Most of the perpetrators (51.4%) were male, and the victims were equally divided to males and females. The ages of the perpetrators ranged from 13 to 61 years, and the ages of the victims ranged between 0 and 17 years. Nearly one-third (32.4%) of the victims were between 5 and 12 years old. Six neonaticides and four infanticides were reported. Adolescents were the least affected age group (five victims, 14.7%). When the victim was younger than 1 year old, the perpetrator was almost always female, whereas older children and adolescents were mostly killed by a male perpetrator (Table 2).

Most perpetrators (54.5%) had a previous psychiatric history and in many cases, they committed (33.3%) or attempted (15.2%) suicide after the homicide.

In most cases, the victims were killed by one parent (twenty-six cases) or both parents (one case), and in five cases the perpetrator was a close family member (brother, uncle, or aunt). Incidents with a perpetrator (usually parent) of the opposite sex (59.4%) of the victim slightly outweighed incidents with a perpetrator of the same sex. In one case, the perpetrator was the aunt of the victim, and she was also the caregiver as the child had been separated from its biological parents by social services. In only one case, the perpetrator was the half-brother of the victim.

**Figure 2:**
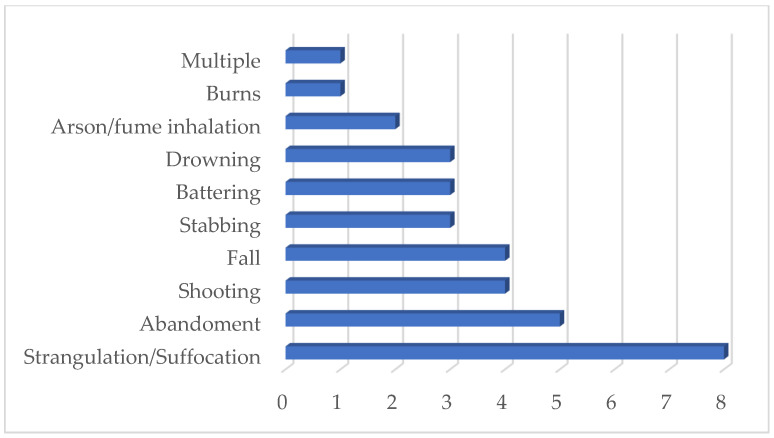
Methods of intrafamilial child/adolescent homicides in Greece during the period 2010–2020.

The most common method of homicide was strangulation, usually combined with suffocation (25%), followed by abandonment (15.6%) (Figure 2). A remarkable case was the death of a little boy who was killed by both of his parents. The boy died due to severe burns on over 70% of his total body surface area caused by his parents, who poured hot water on him in the context of domestic violence. There was also a case in which two twin boys died due to fume inhalation after their house was set fire to by their father, who had locked them in. The father died in the same fire as he had also locked himself in with the children to commit suicide. Furthermore, there was a similar case of paternal filicide in which the father used multiple homicide methods. He stabbed his 17-year-old daughter and then set fire to the house after he locked himself and his daughter in. They were both pulled from the fire alive but the girl died in hospital, while the father eventually survived.

The most reported motives were spousal revenge (nine incidents, 26.5%) and psychotic disorders (nine incidents, 26.5%). Spousal revenge was reported only in paternal filicides in which the father killed the child(ren) as an act of revenge against his ex-wife. On the other hand, unwanted pregnancy seemed to be the motive (six incidents, 17.6%) reported in maternal filicides against neonates. There were also four “accidental” filicides (11.8%) because of extreme domestic violence. A few intrafamilial child homicides were presented as altruistic filicides (or extended suicides) (three incidents, 8.8%). There was also a filicide reported as an “honor” killing. It was the killing of a 17-year-old girl by her father, who considered that her intimate relationship with a boy was a dishonor to his family. The father had a known psychiatric history, and he had been recently discharged from a psychiatric clinic where he was hospitalized.

## 4. Discussion

The act of parents killing their own children, often referred to as filicide or familicide, represents a form of homicide that garners significant attention from the media, particularly because of its rarity and abominable nature. Media interest seems to be greater when the perpetrator of the filicide is a parent with an atypical profile, when the case has temporal proximity with other cases [27], or when it appears that health or social services could have taken measures to prevent the homicide [28].

Especially for maternal filicides, there is a practice of narrating filicide as a crime committed by a contemporary “Medea”, a “monstrous” mother, which is reflected in the media coverage of these homicides [29,30,31]. On the other hand, media often present paternal filicides as the result of domestic violence stemming from social conditions and the weaknesses of the welfare state and/or an indication of the male perpetrator’s extraordinary mental illness [32].

While global estimates indicate that deaths resulting from filicide are relatively rare, achieving an accurate assessment of the frequency of filicide has been challenging due to inconsistent data and accessibility issues. Furthermore, it is impossible to ascertain the true rate of filicides as some perpetrators may have concealed their actions successfully [33]. Notably, to the best of our knowledge, no prior study has specifically investigated intrafamilial child homicide in Greece.

### 4.1. Neonaticide–Infanticide

Depending on the age of the child, there are subcategories of filicide. Filicide within the first 24 h of the life of the child is called “neonaticide”, a term coined by Resnick [34,35]. A filicide by a mother within the first year of life is called “infanticide”. We identified six neonaticides (17.6%) and four infanticides (11.8%). Although middle-childhood-aged children (5–12 years old) are considered more “protected” from homicide than their younger (0–4 years old) or older (13–17 years old) counterparts, in our sample, nearly one-third of the victims (11/34, 32.4%) belonged to this age group.

The term “infanticide” also has medicolegal implications. It mainly applies to the homicide of a child under the age of 12 months by a mother who has not fully recovered from the effects of pregnancy, childbirth, and lactation and who suffers from postpartum psychiatric disorders [36]. In the late nineteenth century, Esquirol and Marce, two French psychiatrists, described for the first time a causal relationship between pregnancy and childbirth and the development of a mental disorder in the mother [37]. This medical perspective regards the crime of infanticide as a manifestation of psychiatric rather than moral issues, thereby justifying a more compassionate legal response grounded in scientific evidence. Presently, numerous countries have implemented legal frameworks that mitigate the penalties imposed on mothers found guilty of committing infanticide [38].

In the Greek Criminal Code, a mother who commits infanticide is described in Section 303 as “A mother who intentionally killed her child during or after childbirth, but while her body was still disturbed by the effect of giving birth, shall be punished by imprisonment for up to ten years”. Infanticide can only be committed by the mother while her body is disarranged by the effects of pregnancy and childbirth, as evidenced by psychiatric expertise presented at trial. Therefore, the mother receives a more lenient punishment. Thus, the killing of an infant by the father or another relative does not constitute an offense of infanticide but an offense of homicide, which carries even the penalty of life imprisonment. Similarly, a mother who kills her child older than 12 months no longer receives lenient criminal treatment.

In our study, it is notable that in over one-third (6/15 cases, 40%) of maternal filicide cases, the victims were neonates (<24 h of life) and 60% (9/15 cases) were children under 12 months of age (including neonates). On the other hand, in cases of paternal filicides, most of the victims (10/12 victims, 83.3%) were 4 years of age and older. Indeed, statistically significant differences were shown regarding the age (but not the sex) of the victim between male and female perpetrators. Neonates and infants (<12 months old) were mostly killed by their female caregivers–mothers, while the majority of children one year of age or older were killed by their fathers. The above findings are in concordance with previous studies that showed that fathers almost never commit neonaticide [39] and they kill older children more frequently compared to mothers, who mostly kill infants [26,36,40].

### 4.2. Factors Affecting Filicides

Factors that have been reported to affect filicides are violent parental behavior, diagnosed or undiagnosed mental health problems of one or both parents, poverty, unemployment, marginalization, parental substance abuse, and criminal behavior [41,42,43]. The prevalence of ingrained prejudices within traditional cultures which might be questioned by the existence of children represents another significant factor to consider [41]. For instance, in South Asia and China, a culture of “son preference” still exists. In countries like China or India, the ratio of male to female children is considered by some researchers to be unnaturally higher than in the rest of the world [44]. In our research, it is quite remarkable that among the six neonates who were killed by their mothers, only one was female (20%). Furthermore, in many societies, children have been subjected to harm or death when they were regarded as an excessive burden or when their parents lacked the necessary resources to provide for their upbringing. For example, in poor regions of Japan where it was difficult to raise more than one or two children, infanticide or abandonment was culturally sanctioned and, consequently, employed as a method of birth control [45].

### 4.3. Maternal Filicide

Although there are cases of both mothers and fathers who kill their children, maternal homicides tend to attract more media attention and cause more public outcry. Mothers who have murdered their children are usually portrayed by the media as satanic or insane. According to the cultural conceptions of the female nature, violence is generally considered incompatible with the female nature. A violent mother who kills her child does not fulfill the role of the typical woman, so she must be satanic or mentally disturbed. It is generally very difficult for someone to realize that a mentally healthy mother is capable of such an act. However, in cases of neonaticides and infanticides, mothers who are mentally disturbed by the effects of childbirth are probably underdiagnosed. Research has indicated that mothers who commit filicide are more likely to have experienced childhood physical abuse and to exhibit signs of mental illness [33]. The female perpetrators of filicide frequently fit the profile of low-income, socially isolated, full-time caretakers who encountered domestic abuse or other relationship-related challenges. Many of these female perpetrators hailed from disadvantaged socioeconomic backgrounds and bore the primary responsibility for childcare duties. Many of these female offenders have been reported to suffer from depression, suicidal thoughts, or psychosis [46]. In our sample, only 43.8% of the mothers who killed their children had a known psychiatric history.

### 4.4. Paternal Filicide

While historically, filicide has been mainly connected with mothers, fathers have become increasingly likely to commit this type of homicide [2,25,47]. We identified 16 cases of maternal filicide, 11 of paternal filicide, and 1 case in which both parents killed their 3-year-old boy. In general, there is limited literature available on paternal filicide [35]. Quite frequently, the act of a filicide by a father is followed by completed or attempted suicide [11,48,49,50]. Fathers, compared to mothers, have been reported to experience more often unstable marriages or to be separated when they commit their crime. They have also been shown to kill older and male children [33,42]. Finally, they often exhibit characteristics such as aggressive behavior toward their partners, alcohol addiction, personality disorders, and jealousy toward their partners [51].

### 4.5. Motives of Filicide

To understand the motives of a parent who kills her/his own child, multiple filicide classification systems have been developed. Initially, they were based on filicidal crimes performed by mothers [34,52]. Later, more recent filicide classification systems also took into account the motivations of male perpetrators [25,26,52,53]. Bourget and Bradford’s system encompasses four categories.

The first category entails pathological filicide, which includes altruistic filicides, extended suicides, and psychotic filicides. In altruistic filicides, the act of murdering a child is motivated by the intent to relieve the child of either genuine or perceived suffering. In extended suicides, the child is considered an extension of the parent, whereas in psychotic filicides, the perpetrator is motivated by psychotic symptoms.

The second category of filicide refers to deaths as the result of child abuse in the form of physical maltreatment or neglect (domestic violence). Occasionally, these types of deaths are categorized as accidents under the assumption that child homicide was not intentional. Step-parents are disproportionately involved in this classification as they tend to exhibit a higher tendency to mistreat their nonbiological children and invest less in their relationships [54]. Munchausen by proxy syndrome belongs in the above category [55,56].

The third category, neonaticide, is the killing of a newborn because the mother denies that she is pregnant, she is afraid of the discovery of the pregnancy (an unwanted child), or she believes that the child is stillborn. Finally, the fourth category includes retaliatory filicides in which the perpetrators are motivated by revenge toward their partner, suffering from so-called “Medea syndrome” [25,26].

In our study, the motive was spousal revenge in 21.1% of cases and the birth of an unwanted child in 18.2% of cases. The deaths were due to an accident as the result of an extreme form of domestic violence in 12.1% of the cases, whereas pathological filicides accounted for 18.2% of the cases (with psychotic filicides occurring in 9.1% and “altruistic” filicides–extended suicides occurring in 9.1% of the cases).

### 4.6. Methods of Filicide

The most common method of homicide in our study was strangulation (usually combined with suffocation) (23.5%), followed by abandonment (14.7%). Shooting with a firearm, throwing from a height, stabbing, battering, arson (death due to the inhalation of fumes) and burning are methods also mentioned in previous studies on filicides. In a very large study including 94,146 filicide arrests in the USA over a 32-year period, it was found that the most common killing methods included beating with hands and feet, strangulation, asphyxiation, drowning, and throwing from a height—defenestration [40]. In a binational study in Austria and Finland of 120 filicide perpetrators who committed their crimes during the period 1995–2005, arson, beating, negligence, drowning, poisoning, shooting, stabbing, strangulation, and suffocation were mentioned as the methods used [47].

A special mention of smothering/suffocation should be made because of its potential to be used by a perpetrator to conceal a homicide. Smothering may leave few or even no physical findings on victims with a reduced capability to defend themselves. This especially applies in to infants, although even older children have been reported as victims of repeated acts or of a single fatal act of smothering. Indicative findings may include ecchymoses and nail marks around the mouth, the nose, and the rest of the face, foreign material inside the nose and throat, and injuries on the rest of the body, as well as petechiae of the face and conjunctivae. However, the absence of the above findings cannot exclude the possibility of child homicide via suffocation. Moreover, histological examinations are rarely demonstrative, as intrapulmonary hemorrhages and/or intra-alveolar hemorrhages, which may be grounds for suspicion, are not present in all cases of death due to suffocation [56,57].

It is worth noting the case of Wanetta Hoyt who, on 22 April 1995, was convicted of the murder of five of her children via suffocation. The death of these children about 20 years earlier had been attributed to “sudden infant death syndrome” (SIDS). Wanetta Hoyt confessed to police having smothered her children using a pillow, towel, and her shoulder because of their crying. Two of the children had been experiencing recurrent near-death episodes of apnea while at home with their mother but never when they were monitored in a hospital [57]. Child death due to suffocation after repeated near-death episodes of apnea or a single event inflicted by the mother has also been discussed in the context of Munchausen syndrome by proxy [57,58]. The differentiation between sudden infant death syndrome (SIDS) may be impossible via an autopsy alone, and this may apply even to the deaths of older children. A thorough assessment of the circumstances and the medical records in combination with autopsy findings is crucial for revealing the cause and manner of death in such cases [57,58,59,60,61].

Because of the above problems, it is probable that some deaths of children due to suffocation might have erroneously been classified as natural, especially in the absence of adequate information. On the other hand, some fatal cases of domestic violence (homicides) against children have been disguised and recorded as accidents, thus leading to an overestimation of the true prevalence of intrafamilial child homicides.

### 4.7. Limitations

Prior studies in the United States [62,63], Italy [64], The Netherlands [65], and Greece [48] employed newspaper surveillance and/or web search queries to estimate the prevalence of various types of homicides, including those that capture media attention, such as homicide–suicide incidents.

The method described above has its limitations and utility, as discussed elsewhere [48]. The information, including psychiatric diagnoses and motives, reported via news websites can be oversimplified or distorted. Filicidal parents rarely express clear motives [30,31], and journalists may lack awareness of certain case aspects that are either intentionally concealed or emerge later during authorities’ investigations. While data from investigation files or forensic reports would offer greater reliability and precision, collecting such data from various sources across the country over an extended period was not feasible. Nonetheless, this research query provides insight into the state of child homicides by family members in Greek society over the past decade, even though the results may represent a conservative estimate. This phenomenon is particularly alarming in Greece, where the well-being of children is closely tied to the role of the family. Implementing a national tracking system in the future would enhance the precision of the recording and assessment of the situation.

## 5. Conclusions

Over the past 11 years, the intrafamilial child homicide rate in Greece approximated 0.15 cases per year per 100,000 persons, in line with international data. The majority of the perpetrators were male, and the victims were evenly distributed between males and females. Male perpetrators tended to target older children compared to female perpetrators. More than half of the perpetrators had a documented psychiatric history. The most frequently employed method of homicide was strangulation, often combined with suffocation, followed by abandonment. The most commonly reported motives were spousal revenge and psychotic disorders. This study has certain limitations as it relied on web search queries. Information, particularly psychiatric diagnoses and reported motives, found on news websites, may be oversimplified or distorted. Obtaining data from investigation files or forensic reports could have provided more reliable and precise results. However, collecting such data from various services across the country over an extended period of time was not feasible.

Targeted efforts for the timely recognition of potential victims and perpetrators and a high level of awareness for identifying even covert intrafamilial child homicides are of the utmost importance for the prevention of this dreadful phenomenon. Social assistance and child protection play vital roles when parents struggle to maintain control over their lives while caring for their children. There is an urgent need for the establishment of centers at which experts from various fields can collaborate to conduct comprehensive investigations into hidden child homicides. While intrafamilial child homicide is rare, recording it meticulously is a crucial step, even though identifying specific trends and patterns can be challenging. The findings of this study shed light on the prevalence of child and adolescent homicides in Greece over the past decade, also highlighting the need for improved data collection and coordination among law enforcement agencies.

## Figures and Tables

**Table 1:** The sociodemographic characteristics of the study sample.

Variables	Subgroups	N (%)
**Perpetrators (N = 33)**		
Sex	Male	18 (51.4%)
	Female	17 (48.6%)
Age Mean age: 36.58 ± 13.04 years	0–18 years	1 (3.0%)
	19–35 years	16 (48.5%)
	36–55 years old	13 (39.4%)
	>55 years old	3 (9.1%)
Psychiatric history	Existent	18 (54.5%)
	Non-existent	12 (36.4%)
	Unknown	3 (9.1%)
Suicide	Completed	11 (33.3%)
	Attempted	5 (15.2%)
	No	17 (51.5%)
**Victims (N = 34)**		
Sex	Male	17 (50.0%)
	Female	17 (50,0%)
Age	Neonates (<24 h of life)	6 (17.6%)
	Infants (<12 months)	4 (11.8%)
	Pre-school children (1–4 years)	8 (23.5%)
	Middle-childhood-aged children (5–12 years)	11 (32.4%)
	Adolescents (13–17 years)	5 (14.7%)
**Other information**		
Sex of the perpetrator vs. victim (per victim)	Same	14 (41.2%)
	Opposite	19 (55.9%)
	Both (multiple perpetrators)	1 (2.9%)
Relationship between perpetrator and victim (per victim)	Parent	27 (79.4%)
	Uncle/aunt	5 (14.7%)
	Brother (half-brother)	2 (5.9%)
Method of homicide (per victim)	Strangulation/suffocation	8 (23.5%)
	Abandonment	5 (14.7%)
	Shooting (firearm)	4 (11.8%)
	Fall	4 (11.8%)
	Stabbing	3 (8.8%)
	Battering	3 (8.8%)
	Drowning	3 (8.8%)
	Arson/fume inhalation	2 (5.9%)
	Burns	1 (2.9%)
	Multiple	1 (2.9%)
Motivation (per perpetrator)	Spousal revenge	9 (26.5%)
	Psychotic filicide	9 (26.5%)
	Unwanted child	6 (17.6%)
	Accidental (domestic violence)	4 (11.8%)
	“Altruistic” filicide—extended suicide	3 (8.8%)
	Honor killing	1 (2.9%)
	Other/unspecified	2 (5.9%)

**Table 2:** Associations of characteristics according to the perpetrators’ sex.

Variables	Subgroups	Male	Female	*p*
Psychiatric history (*n* = 31)	Existent	11 (61.1%)	7 (38.9%)	0.190 ^‡^
	Non-existent	3 (27.3%)	8 (72.7%)	
	Unknown	1 (50.0%)	1 (50.0%)	
Suicide (*n* = 31)	Completed	7 (63.6%)	4 (36.4%)	0.555 ^‡^
	Attempted	2 (40.0%)	3 (60.0%)	
	No	6 (40.0%)	9 (60.0%)	
Victim’s sex (*n* = 33)	Male	7 (43.8%)	9 (56.3%)	0.494 ^†^
	Female	10 (58.8%)	7 (41.2%)	
Victim’s age (*n* = 33)	Neonates (<24 h of life)	0 (0.0%)	6 (100.0%)	0.022 ^‡^
	Infants (<12 months)	1 (25.0%)	3 (75.0%)	
	Pre-school children (1–4 years)	5 (71.4%)	2 (28.6%)	
	Middle-childhood-aged children (5–12 years)	8 (72.7%)	3 (27.3%)	
	Adolescents (13–17 years)	3 (60.0%)	2 (40.0%)	

Values were referred to absolute and relative frequencies (%). *p*-values were calculated after conducting ^†^ Fisher’s exact test or a ^‡^ Monte Carlo simulation.

## Data Availability

All data generated or analyzed during this study are included in this published article or are available from the corresponding author upon reasonable request.
